# Estimated HIV Incidence in the United States, 2006–2009

**DOI:** 10.1371/journal.pone.0017502

**Published:** 2011-08-03

**Authors:** Joseph Prejean, Ruiguang Song, Angela Hernandez, Rebecca Ziebell, Timothy Green, Frances Walker, Lillian S. Lin, Qian An, Jonathan Mermin, Amy Lansky, H. Irene Hall, for the HIV Incidence Surveillance Group

**Affiliations:** 1 Division of HIV/AIDS Prevention, National Center for HIV, Viral Hepatitis, STD and TB Prevention, Centers for Disease Control and Prevention, Atlanta, Georgia, United States of America; 2 The Ginn Group, Peachtree City, Georgia, United States of America; National University of Singapore, Singapore

## Abstract

**Background:**

The estimated number of new HIV infections in the United States reflects the leading edge of the epidemic. Previously, CDC estimated HIV incidence in the United States in 2006 as 56,300 (95% CI: 48,200–64,500). We updated the 2006 estimate and calculated incidence for 2007–2009 using improved methodology.

**Methodology:**

We estimated incidence using incidence surveillance data from 16 states and 2 cities and a modification of our previously described stratified extrapolation method based on a sample survey approach with multiple imputation, stratification, and extrapolation to account for missing data and heterogeneity of HIV testing behavior among population groups.

**Principal Findings:**

Estimated HIV incidence among persons aged 13 years and older was 48,600 (95% CI: 42,400–54,700) in 2006, 56,000 (95% CI: 49,100–62,900) in 2007, 47,800 (95% CI: 41,800–53,800) in 2008 and 48,100 (95% CI: 42,200–54,000) in 2009. From 2006 to 2009 incidence did not change significantly overall or among specific race/ethnicity or risk groups. However, there was a 21% (95% CI:1.9%–39.8%; p = 0.017) increase in incidence for people aged 13–29 years, driven by a 34% (95% CI: 8.4%–60.4%) increase in young men who have sex with men (MSM). There was a 48% increase among young black/African American MSM (12.3%–83.0%; p<0.001). Among people aged 13–29, only MSM experienced significant increases in incidence, and among 13–29 year-old MSM, incidence increased significantly among young, black/African American MSM. In 2009, MSM accounted for 61% of new infections, heterosexual contact 27%, injection drug use (IDU) 9%, and MSM/IDU 3%.

**Conclusions/Significance:**

Overall, HIV incidence in the United States was relatively stable 2006–2009; however, among young MSM, particularly black/African American MSM, incidence increased. HIV continues to be a major public health burden, disproportionately affecting several populations in the United States, especially MSM and racial and ethnic minorities. Expanded, improved, and targeted prevention is necessary to reduce HIV incidence.

## Introduction

The Centers for Disease Control and Prevention (CDC) maintains an HIV surveillance system in which all states and U.S. territories submit data on reported diagnoses of HIV. The data are de-duplicated at CDC both within and across states, and the reported case counts are adjusted for reporting delay to estimate the number of new diagnoses of HIV infection and AIDS, annually [1]. These estimates have been used to track the HIV epidemic in the United States, but because HIV diagnosis can occur at any point during the long latency between infection and symptom development in HIV disease the estimates have been limited by their reliance on HIV testing and reporting practices. In response to these limitations, CDC, in conjunction with a number of state and local health departments, implemented national HIV incidence surveillance, using a serologic marker of recent HIV infection to classify new diagnoses of HIV infection as either recent or long-standing [2]. Additional data on history of HIV testing and antiretroviral use are collected to determine sampling weights for estimation of the number of new HIV infections in the United States, both diagnosed and undiagnosed [3]. Based on data from HIV incidence surveillance, Hall and colleagues developed the first national HIV incidence estimate based on a direct measure of recency of infection [4]. It was estimated that in 2006 approximately 56,300 (95% confidence interval [CI]: 48,200–64,500) individuals were infected with HIV.

HIV surveillance is a dynamic system with additional data continually reported to state surveillance systems, and estimates of HIV diagnoses and incidence are updated to reflect these newly available data, science, and programmatic considerations. For example, the incidence estimation model is sensitive to sudden changes in HIV testing patterns which could influence estimates of HIV incidence [4] if recommendations for routine HIV testing [5] are fully implemented. In addition, the continued refinement of the method for estimating the mean recency period for the BED HIV-1 Capture Enzyme Immunoassay (BED; the assay that currently serves as the serologic marker of recent infection) [6] and for modeling the recency period distribution [7] has allowed for improvement of the method of modeling HIV incidence [3]. We updated the earlier estimate of HIV incidence for 2006 based on additional data and methodological refinements, and extended the previous results with estimates for the years 2007, 2008, and 2009.

## Methods

Since 1982, all 50 U.S. states and the District of Columbia have reported AIDS cases to CDC. In 1994, CDC began receiving reports of diagnosed HIV infection from the 25 states with confidential name-based HIV reporting. As of 2008, all 50 states, the District of Columbia and 5 territories submit de-identified data on reported diagnoses of HIV infection to CDC.

Since 2004, CDC has funded selected states and localities to conduct HIV incidence surveillance by (1) submitting remnant diagnostic HIV-positive blood specimens for testing using the serologic testing algorithm for recent HIV seroconversion (STARHS) [8] and (2) collecting supplemental data on history of HIV testing and antiretroviral use. STARHS is a two test algorithm in which the first test is used to determine whether an individual is HIV-positive on a standard enzyme-linked immunoassay (EIA) test. Currently within STARHS the EIA is followed by the BED which measures the concentration of anti-HIV IgG to total IgG in a sample. The result is reported as a standard optical density (SOD), a continuous measure that describes the relative concentration of anti-HIV IgG. If the SOD for a given sample is over a threshold predetermined to define a “long-standing” infection then the infection is deemed no longer recent. The period of time during which the SOD is below this threshold is termed the recency period, denoted by W. Its associated survival function, Pr(W>t) is denoted by S_W_(t).

HIV incidence surveillance was designed to take advantage of national HIV surveillance by incorporating needed information on history of HIV testing and antiretroviral use and STARHS result data into routine case reporting in the states and cities that receive funding to conduct HIV incidence surveillance. From 2004 through 2007, 34 surveillance areas, including 27 state/territorial health departments and 7 city/county health departments, participated in HIV incidence surveillance. Since 2008, HIV incidence surveillance areas have included 25 health departments, including 18 state, and 7 city/county health departments. HIV incidence surveillance areas collect information on the self-reported date of first HIV antibody positive test, date of most recent HIV antibody negative test, number of negative HIV tests in the two years before testing positive, and antiretroviral usage (including beginning and ending dates, if applicable). In addition, HIV incidence surveillance coordinators in these areas collaborate with public and commercial laboratories to locate and ship remnant diagnostic blood specimens for testing using STARHS at a single national laboratory [2]. The BED has been used with STARHS in the United States since 2005 to classify new diagnoses of HIV infection as either recent or long-standing.

Despite the relative advantage of using the existing HIV surveillance system and its resources to implement HIV incidence surveillance, health departments face logistical challenges in securing remnant HIV-positive blood specimens from multiple laboratories for use in STARHS. As a result of these challenges, remnant diagnostic blood specimens are frequently unavailable for STARHS. Although the percentage of cases reported to national HIV surveillance with a STARHS result has increased annually in all HIV incidence surveillance areas, in order to examine the temporal trend in incidence from 2006 through 2009, only those areas that met certain minimum criteria—15% completeness of STARHS results (as in previous analyses), confidential name-based reporting of HIV cases, and continuous implementation of HIV incidence surveillance throughout the entire analysis period—were included in this analysis. Those surveillance areas included 16 states (Alabama, Arizona, Colorado, Connecticut, Florida, Indiana, Louisiana, Michigan, Mississippi, New Jersey, New York, North Carolina, South Carolina, Texas, Virginia, and Washington) and 2 cities (Chicago and Philadelphia). While the minimum inclusion criterion was 15% completeness of STARHS results, the lowest level of completeness for any area was 17% in 2006, 20% in 2007, 27% in 2008 and 22% in 2009.

We included in the analysis new diagnoses of HIV infection (regardless of the stage of disease) among individuals aged 13 years and older at diagnosis in the years 2006 through 2009, with a residence at diagnosis in one of the aforementioned areas, and reported to CDC through June 2010. Data on STARHS results and history of HIV testing and antiretroviral use included data reported to CDC through January 2011 for these cases.

We estimated HIV incidence using the stratified extrapolation method described by Karon and colleagues [3], which is based on concepts borrowed from sample survey methodology. The total number of diagnosed and undiagnosed HIV infections in a single year is estimated based on the observed number of new HIV diagnoses classified as recent infections using STARHS and the estimated probability that a new HIV infection would be diagnosed within the STARHS recency period (and thus classified as a recent infection). Based on additional available data, we modified two major components of the previously published method: (1) the method for estimating the probability of being detected in the STARHS recency period, and (2) the method for addressing missing information on transmission category.

Previously, the probability of being detected in the STARHS recency period (*P*) was simply calculated as the product of two probabilities: (1) that of being tested within one year after infection (*P_1_)*, and (2) that of an infection being classified recent using STARHS, given a test within one year after infection (*P_w_*). The previous method was simple because the second probability could be approximated by the mean STARHS recency period. However, this simple estimate is subject to bias in two instances: (1) when the time of HIV testing within one year after infection is not uniformly distributed in that one-year interval, or (2) when the probability of an individual having a STARHS recency period longer than one year is not close to zero. The first assumption is violated in population groups with frequent HIV testing and leads to an artificial upper limit on the calculated probability of being detected in the STARHS recency period equal to *P_w_* because *P_1_* cannot be greater than 1. With the additional data available subsequent to the initial 2006 HIV incidence estimate, the extent of these biases became clear, as both the impact of testing frequency, and the probability of an individual having a STARHS recency period longer than one year (currently estimated to be 0.049 using the BED) were greater than anticipated in this population. To eliminate these biases we now calculate *P* directly from the testing frequency distribution observed in the population. For repeat testers (i.e., those individuals with a previous negative HIV test), *P* is calculated based on the distribution of *T*, the time from the last negative test to the first positive test. Under the assumption that the infection date is uniformly distributed in the interval [0,T], we have:
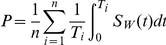
where *S_W_*(*t*) is the probability that someone infected with HIV *t* years ago would have remained ‘recent’ on the BED assay since acquiring HIV (i.e., the STARHS recency period survival function), *n* is the number of repeat testers (people testing positive after a previous negative test), and *T_i_* is the time *T* from the *i*th repeat tester. We used a nonparametric step function to estimate S_W_(t), with a mean of 162 days, based on recently-updated estimates of the recency period distribution [7]. Table S1 displays the probability of having an HIV test within the BED recency period (*P*) for a given number of months between the last negative HIV test and the first positive test (*T*). The average group level probability (*P*) for each of 68 homogeneous demographic groups of repeat testers was used in the estimation of incidence for repeat testers.

For new testers (i.e., those whose first HIV test was positive), the average group-level probability, *P*, is estimated based on the HIV testing rate which is in turn estimated from a competing risk model (time to AIDS diagnosis vs. time to HIV test) and the probability of AIDS at HIV diagnosis (progression to AIDS before a HIV test). Let *X* be the time from HIV infection to the first HIV positive test. Based on Karon et al [3], *X* has an exponential distribution with a scale parameter 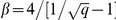, where *q* is the probability of AIDS at HIV diagnosis within the group. Let *W* be the recency period length, *A* the time from HIV infection to AIDS diagnosis (the AIDS incubation period), and assume that *W* and *A* are independent. Then, the expected group-level probability (*P*) is:
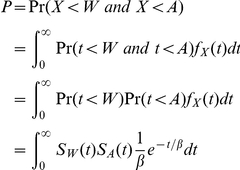
where *S_A_*(*t*) is the survival function for the AIDS incubation period (from HIV infection to AIDS diagnosis) without treatment represented by a gamma distribution with a shape parameter α_A_ = 2 and a scale parameter β_A_ = 4. Table S2 displays the probability of having an HIV test within the BED recency period (*P*) that is assigned to each new tester in a group based on the group's proportion of cases diagnosed with AIDS at the time of the HIV diagnosis (*q*).

The other major improvement in the estimation procedure is the approach to handling missing transmission category. Previously, cases with missing transmission category were separated from those with known transmission category in the imputation and incidence estimation procedures. The estimated incidence in this group was then redistributed to other risk groups consistent with methods previously used to address missing risk factor information in national HIV surveillance data. [9]. Since the 2006 estimate, a more accurate, multiple imputation method for assigning mode of transmission based on observed data, rather than historical trends have been developed and applied to the national surveillance system [10]. We have applied those same methods in the revised incidence estimation model, imputing missing transmission category values. The imputed transmission category values were then used in imputing STARHS results and testing history information and in the subsequent estimation procedures, thus eliminating the additional step of redistributing the incidence within the missing transmission category group to other transmission category groups.

Following the previous model, incidence estimates were adjusted to account for those newly diagnosed but not yet reported HIV cases. Finally, the estimated incidence within the areas contributing data for estimation was extrapolated to the remaining U.S. areas to obtain a national estimate by applying the group-specific ratio of HIV incidence to diagnoses of AIDS within the areas contributing data to the number of new diagnoses of AIDS in the remaining U.S. areas. Population denominators for the calculation of rates were based on intercensal estimates for 2006—2009 obtained from the United States Census Bureau [11]. Annual differences in HIV incidence overall and within groups were determined using the z-test. To eliminate the correlation between annual incidence estimates, the uncertainty associated with the STARHS recency period distribution estimate (a common factor within each year's incidence estimate) was removed from the calculation of the z-score. We also calculated the estimated annual percentage change (EAPC) in the estimated number of new HIV infections by fitting a logistic regression model to the natural logarithm of the incidence estimate using calendar year as the regressor [12].

## Results

The total number of persons aged 13 years and older diagnosed with HIV infection in the surveillance areas for the years 2006–2009 and reported through June 2010 was 29,279, 29,943, 28,831, and 27,040, respectively. Adjustment for reporting delay brought the totals to 30,702, 31,883, 31,357, and 31,162 respectively. Among individuals not diagnosed with AIDS within 6 months of HIV diagnosis, the number with BED results by year was 6,096 (31%) for 2006, 7,615 (37%) for 2007, 8,863 (44%) for 2008 and 9,615 (50%) for 2009. Among individuals who had a remnant diagnostic specimen tested with the BED in 2006, a higher percentage were black/African American, men who have sex with men (MSM) and in the youngest age group when compared to the distribution of new diagnoses. In 2007 a higher percentage were women, black/African American and in the youngest age group, and a lower percentage were injection drug users. In 2008 and 2009, a higher percentage were black/African American and in the youngest age group (Table S3). Of those without a diagnosis of AIDS at or within six months of HIV diagnosis, after imputation, the percent classified recent by year was 31%, 33%, 31%, and 30%. By year, 10,954 (37%), 13,322 (44%), 14,031 (49%), and 14,805 (55%) individuals had information on whether they had had a previous HIV-negative test, and after imputation 17,033 (58%), 16,533 (55%), 16,465 (57%), and 15,237 (56%) were classified as repeat testers, respectively. Among repeat testers, the percentage with *T* less than or equal to 12 months was 43%, 41%, 42%, and 43% respectively, by year. The proportion of cases with concurrent HIV and AIDS diagnoses was 21%, 19%, 19% and 18%, respectively.

**Figure 1:**
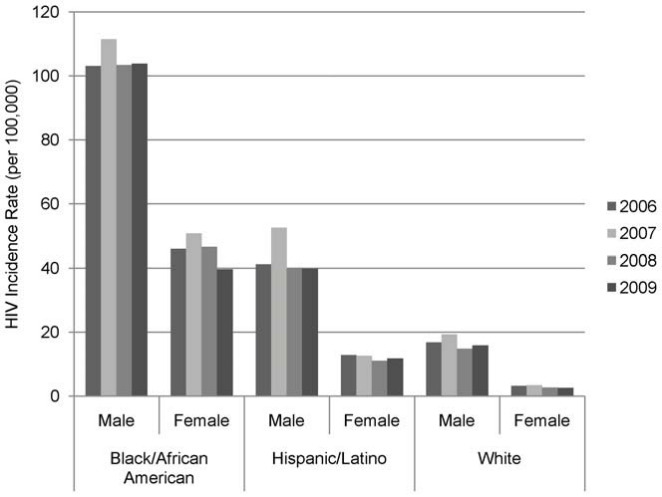
Rate (per 100,000) of new HIV infections by gender and race/ethnicity – United States, 2006–2009. Each year, the highest rate of new infections was in black/African American males. Among females, blacks/African Americans also had the highest rates of new infections annually.

Based on the revised stratified extrapolation approach with a recalculated mean STARHS recency period using the BED of 162 days and using new diagnoses of HIV infection reported through June 2010, an estimated 48,600 individuals aged 13 years or older in the United States were infected with HIV in 2006 (95% CI: 42,400–54,700), with an additional 56,000 (95% CI: 49,100–62,900), 47,800 (95% CI: 41,800–53,800) and 48,100 (95% CI: 42,200–54,000) infected in 2007, 2008 and 2009, respectively. In each year, the most new infections occurred in males (accounting for 75%, 76%, 75%, and 77% of new infections respectively), MSM (56%, 58%, 56%, and 61%), and blacks/African Americans (44%, 42%, 46%, and 44%). The rate of new infections overall for 2006 through 2009 was estimated to be 19.8 (95% CI: 17.3–22.2), 22.5 (95% CI: 19.7–25.3), 19.0 (95% CI: 16.6–21.4), and 19.0 (95% CI: 16.6–21.3) per 100,000 individuals, respectively. Blacks/African Americans and Hispanics/Latinos experienced the heaviest impact of the epidemic with rates that were 7.4 and 2.8 times the rate in whites respectively in 2006, 7.1 and 3.0 times the rate in whites in 2007, 8.4 and 3.0 times the rate in whites in 2008, and 7.7 and 2.9 times the rate in whites in 2009 (Table 1). Each year blacks/African American males had the highest rate of new infections, and among women, black/African American women also experienced the highest HIV incidence rates (Figure 1).

**Table 1:** Estimated Incidence of Human Immunodeficiency Virus Infection and Rate per 100,000, 50 U.S. States and the District of Columbia, 2006–2009.

		2006		2007	
Characteristic		Incidence, No. (%) [95% CI]^a^	Rate [95% CI]	Incidence, No. (%) [95% CI]^a^	Rate [95% CI]
Total^b^		48,600 [42,400–54,700]	19.8 [17.3–22.2]	56,000 [49,100–62,900]*	22.5 [19.7–25.3]
Sex	Male	36,200 (75) [31,300–41,100]	30.1 [26.0–34.2]	42,400 (76) [36,900–47,900]*	34.9 [30.3–39.4]
	Female	12,400 (25) [10,400–14,300]	9.8 [8.3–11.4]	13,600 (24) [11,500–15,800]	10.7 [9.0–12.4]
Race/ethnicity	American Indian/Alaska Native	150 (<1) [0–380]	8.3 [0.0–20.9]	210 (<1) [0–520]	11.6 [0.0–28.2]
	Asian	880 (2) [30–1,700]	8.4 [0.3–16.4]	1,200 (2) [330–2,200]	11.5 [3.0–20.0]
	Black/African American	21,200 (44) [18,400–24,000]	72.7 [63.0–82.4]	23,400 (42) [20,400–26,500]	79.2 [68.9–89.6]
	Hispanic/Latino^c^	9,000 (18) [7,400–10,600]	27.6 [22.6–32.5]	11,200 (20) [9,300–13,100]*	33.4 [27.8–39.0]
	Native Hawaiian/Other Pacific Islander	130 (<1) [0–420]	38.8 [0.0–124.5]	190 (<1) [0–890]	54.0 [0.0–258.4]
	White	16,600 (34) [13,800–19,400]	9.8 [8.1–11.5]	18,900 (34) [15,800–22,100]	11.2 [9.3–13.0]
	Multiple races	680 (1) [330–1,000]	25.4 [12.2–38.7]	750 (1) [370–1,100]	27.2 [13.3–41.0]
Age at infection (yr)	13–29	15,600 (32) [13,100–18,000]	21.8 [18.4–25.2]	20,100 (36) [17,200–23,100]*	27.9 [23.8–32.0]
	30–39	14,900 (31) [12,500–17,400]	37.0 [30.9–43.2]	16,700 (30) [14,100–19,400]	41.5 [35.0–48.0]
	40–49	12,600 (26) [10,400–14,700]	27.9 [23.1–32.7]	13,100 (23) [11,000–15,200]	29.3 [24.6–34.1]
	50–99	5,500 (11) [4,300–6,700]	6.2 [4.8–7.6]	6,000 (11) [4,800–7,200]	6.6 [5.2–7.9]
Transmission category	Male-to-male sexual contact	27,000 (56) [23,000–31,000]		32,300 (58) [27,800–36,800]*	
	Injection drug use	5,300 (11) [4,000–6,600]		5,900 (10) [4,500–7,200]	
	Male-to-male sexual contact and Injection drug use	1,900 (4) [1,200–2,700]		1,900 (3) [1,300–2,600]	
	Heterosexual contact^d^	14,300 (29) [11,900–16,600]		15,700 (28) [13,400–18,100]	
	Other^e^	80 (<1) [0–210]		100 (<1) [0–270]	

		2008		2009	
		Incidence, No. (%) [95% CI]^a^	Rate [95% CI]	Incidence, No. (%) [95% CI]^a^	Rate [95% CI]
Total^b^		47,800 [41,800–53,800]**	19.0 [16.6–21.4]	48,100 [42,200–54,000]**	19.0 [16.6–21.3]
Sex	Male	35,600 (75) [30,900–40,300]**	29.0 [25.2–32.9]	36,900 (77) [32,200–41,600]**	29.8 [26.0–33.6]
	Female	12,100 (25) [10,200–14,100]	9.5 [7.9–11.0]	11,200 (23) [9,200–13,100]**	8.6 [7.1–10.1]
Race/ethnicity	American Indian/Alaska Native	220 (<1) [0–480]	11.9 [0.0–26.0]	260 (1) [0–640]	14.0 [0.0–33.9]
	Asian	1,100 (2) [350–1,800]	9.7 [3.1–16.2]	940 (2) [340–1,500]	8.3 [3.0–13.6]
	Black/African American	21,900 (46) 18,900–25,000]	73.2 [63.2–83.3]	21,200 (44) [18,400–24,000]	69.9 [60.6–79.1]
	Hispanic/Latino^c^	9,000 (19) [7,500–10,600]**	26.1 [21.6–30.6]	9,400 (20) [7,800–11,000]	26.4 [22.0–30.9]
	Native Hawaiian/Other Pacific Islander	70 (<1) [0–790]	20.0 [0.0–225.3]	160 (<1) [0–980]	43.7 [0.0–274.9]
	White	14,800 (31) [12,300–17,200]**	8.7 [7.2–10.1]	15,600 (32) [13,000–18,200]**	9.1 [7.6–10.6]
	Multiple races	660 (1) [370–950]	23.1 [12.9–33.4]	520 (1) [250–800]	17.9 [8.4–27.3]
Age at infection (yr)	13–29	18,600 (39) [15,900–21,300]*	25.6 [21.8–29.4]	18,800 (39) [16,100–21,600]*	25.8 [22.1–29.6]
	30–39	13,800 (29) [11,500–16,100]**	34.2 [28.6–39.8]	13,000 (27) [10,900–15,100]**	32.2 [27.0–37.4]
	40–49	10,400 (22) [8,600–12,300]**	23.6 [19.4–27.7]	11,000 (23) [9,100–12,900]	25.1 [20.8–29.4]
	50–99	5,000 (10) [3,900–6,000]	5.3 [4.2–6.4]	5,200 (11) [4,100–6,400]	5.5 [4.3–6.6]
Transmission category	Male-to-male sexual contact	26,900 (56) [23,200–30,600]**		29,300 (61) [25,400–33,200]	
	Injection drug use	5,100 (11) [3,700–6,400]		4,500 (9) [3,100–5,900]	
	Male-to-male sexual contact and Injection drug use	1,200 (3) [710–1,700]		1,300 (3) [790–1,800]	
	Heterosexual contact^d^	14,500 (30) [12,300–16,700]		12,900 (27) [10,800–15,000]**	
	Other^e^	120 (<1) [0–300]		70 (<1) [0–210]	

a CI, Confidence Interval. Confidence intervals reflect random variability affecting model uncertainty but may not reflect model-assumption uncertainty; thus, they should be interpreted with caution.

b Because column totals for estimated numbers were calculated independently of the values for the subpopulations, the values in each column may not sum to the column total.

c Hispanics/Latinos can be of any race.

d Heterosexual contact with a person known to have, or to be at high risk for, HIV infection.

e Includes hemophilia, blood transfusion, perinatal exposure, and risk factor not reported or not identified.

*Indicates significantly different (p<0.05) from the 2006 estimate for the same group.

**Indicates significantly different (p<0.05) from the 2007 estimate for the same group.

From 2006 to 2009 there was no significant change in HIV incidence overall and there was no significant change in incidence in any race/ethnicity group or risk group overall. There was an overall significant increase in HIV incidence from 2006 to 2007 (15%, 95% CI: 3.6%–26.8%; p = 0.006) with increases in men (17%, 95% CI: 3.0%–31.1%; p = 0.01), Hispanics/Latinos (24%, 95%CI: 0.2%–49.7%; p = 0.027), young people 13–29 years old (29%, 95% CI: 8.6%–49.7%; p = 0.002), and MSM (20%, 95% CI: 2.7%–36.8%; p = 0.013). In all of these groups, except young people, the estimated HIV incidence decreased significantly between 2007 and 2008, in each case falling below 2006 levels. In young people aged 13–29 years the estimated incidence of HIV infection decreased in 2008 as compared to 2007, but remained higher than in 2006, and this group was the only group to evidence a statistically significant increase in HIV incidence between 2006 and 2009 (Table 1). Within the youngest age group only black/African American males experienced a statistically significant increase in HIV incidence from 5,300 (95% CI: 4,200–6,400) in 2006 to 7,600 (95% CI: 6,300–8,900) in 2009, a 43% increase (95% CI: 11.6%–75.2%; p = 0.001) (Table 2). HIV incidence was essentially unchanged 2006–2009 in Hispanic/Latino, and white males aged 13–29 (Tables 3 and 4).

**Table 2:** Estimated Number, Percentage, and Rate per 100,000 of New Infections with Associated 95% Confidence Intervals among Blacks/African Americans - United States, 2006–2009.

		2006		2007	
Characteristic		Incidence, No. (%) [95% CI]^a^	Rate [95% CI]	Incidence, No. (%) [95% CI]^a^	Rate [95% CI]
**Male**					
**Age at infection (yr)**	13–29	5,300 (38) [4,200–6,400]	103.2 [82.5–124.0]	7,100 (46) [5,800–8,400]*	136.2 [110.8–161.5]
	30–39	3,700 (26) [2,800–4,600]	154.3 [115.8–192.8]	3,900 (25) [3,000–4,700]	159.6 [122.8–196.4]
	40–49	3,500 (25) [2,500–4,500]	137.4 [97.0–177.8]	2,900 (19) [2,200–3,700]	116.7 [88.3–145.1]
	50–99	1,600 (11) [1,000–2,200]	44.4 [28.4–60.4]	1,600 (10) [1,000–2,100]	42.2 [28.0–56.4]
**Transmission category**	Male-to-male sexual contact	9,000 (64) [7,400–10,700]		10,400 (67) [8,600–12,200]	
	Injection drug use	1,600 (12) [970–2,300]		1,500 (10) [980–2,100]	
	Male-to-male sexual contact and Injection drug use	700 (5) [340–1,100]		540 (4) [240–850]	
	Heterosexual contact^b^	2,700 (19) [1,900–3,500]		3,000 (19) [2,300–3,700]	
**Subtotal** c		14,100 [11,800–16,300]	103.1 [86.7–119.4]	15,500 [13,100–17,800]	111.5 [94.8–128.1]
**Female**					
**Age at infection (yr)**	13–29	2,200 (30) [1,600–2,800]	42.5 [30.5–54.4]	3,000 (37) [2,300–3,700]*	57.8 [44.7–70.9]
	30–39	2,200 (31) [1,600–2,800]	82.0 [61.0–103.0]	2,300 (28) [1,600–2,900]	84.2 [61.0–107.3]
	40–49	1,900 (26) [1,300–2,400]	63.9 [45.6–82.1]	1,800 (23) [1,300–2,300]	63.0 [44.8–81.1]
	50–99	920 (13) [550–1,300]	19.1 [11.4–26.8]	940 (12) [570–1,300]	18.8 [11.5–26.1]
**Transmission category**	Injection drug use	1,100 (16) [700–1,500]		1,400 (17) [830–1,900]	
	Heterosexual contact^b^	6,000 (84) [4,900–7,100]		6,600 (83) [5,400–7,900]	
**Subtotal** c		7,100 [5,900–8,400]	46.0 [38.0–54.0]	8,000 [6,600–9,400]	50.8 [42.0–59.6]
**Total** c		21,200 [18,400–24,000]	72.7 [63.0–82.4]	23,400 [20,400–26,500]	79.2 [68.9–89.6]

		2008		2009	
		Incidence, No. (%) [95% CI]^a^	Rate [95% CI]	Incidence, No. (%) [95% CI]^a^	Rate [95% CI]
**Male**					
**Age at infection (yr)**	13–29	7,300 (50) [6,000–8,700]*	139.0 [113.3–164.7]	7,600 (51) [6,300–8,900]*	143.2 [118.3–168.0]
	30–39	3,300 (23) [2,400–4,100]	134.9 [100.7–169.2]	3,400 (23) [2,600–4,300]	139.9 [104.2–175.6]
	40–49	2,700 (18) [2,000–3,400]	106.5 [78.8–134.2]	2,600 (18) [1,900–3,300]	104.3 [76.1–132.5]
	50–99	1,300 (9) [820–1,700]	33.0 [21.3–44.7]	1,200 (8) [770–1,600]	29.3 [19.2–39.4]
**Transmission category**	Male-to-male sexual contact	9,800 (68) [8,200–11,500]		10,800 (73) [9,100–12,500]	
	Injection drug use	1,500 (11) [910–2,200]		1,200 (8) [610–1,700]	
	Male-to-male sexual contact and Injection drug use	350 (2) [160–540]		360 (2) [140–570]	
	Heterosexual contact^b^	2,800 (19) [2,100–3,500]		2,400 (17) [1,800–3,100]	
**Subtotal** c		14,500 [12,400–16,700]	103.4 [87.9–119.0]	14,800 [12,600–16,900]	103.9 [88.8–119.0]
**Female**					
**Age at infection (yr)**	13–29	2,500 (33) [1,900–3,000]	47.6 [36.8–58.4]	2,300 (36) [1,700–2,800]	43.8 [33.4–54.2]
	30–39	2,300 (32) [1,700–2,900]	87.2 [64.5–109.9]	1,700 (27) [1,200–2,200]	63.5 [46.0–81.0]
	40–49	1,600 (22) [1,200–2,100]	57.3 [40.5–74.2]	1,400 (22) [990–1,900]	50.5 [34.8–66.2]
	50–99	950 (13) [570–1,300]	18.5 [11.0–25.9]	960 (15) [570–1,300]	18.0 [10.6–25.3]
**Transmission category**	Injection drug use	1,100 (15) [610–1600]		940 (15) [450–1,400]	
	Heterosexual contact^b^	6,300 (85) [5,200–7,400]		5,400 (85) [4,500–6,400]	
**Subtotal** c		7,400 [6,100–8,700]	46.6 [38.3–54.8]	6,400 [5,300–7,500]**	39.7 [32.7–46.8]
**Total** c		21,900 [18,900–25,000]	73.2 [63.2–83.3]	21,200 [18,400–24,000]	69.9 [60.6–79.1]

a CI, Confidence Interval. Confidence intervals reflect random variability affecting model uncertainty but may not reflect model-assumption uncertainty; thus, they should be interpreted with caution.

b Heterosexual contact with a person known to have, or to be at high risk for, HIV infection.

c Because column subtotals and totals for estimated numbers were calculated independently of the values for the subpopulations, the values in each column may not sum to the column subtotal or total.

*Indicates significantly different (p<0.05) from the 2006 estimate for the same group.

**Indicates significantly different (p<0.05) from the 2007 estimate for the same group.

**Table 3:** Estimated Number, Percentage, and Rate per 100,000 of New Infections with Associated 95% Confidence Intervals among Hispanics/Latinos^a^ - United States, 2006–2009.

		2006		2007	
Characteristic		Incidence, No. (%) [95% CI]^b^	Rate [95% CI]	Incidence, No. (%) [95% CI]^b^	Rate [95% CI]
**Male**					
**Age at infection (yr)**	13–29	2,600 (37) [1,800–3,400]	37.6 [26.3–49.0]	3,500 (38) [2,600–4,400]	49.6 [36.5–62.7]
	30–39	2,400 (34) [1,600–3,100]	61.6 [42.4–80.8]	3,100 (34) [2,200–4,000]	77.4 [55.2–99.6]
	40–49	1,500 (21) [890–2,100]	51.6 [30.8–72.4]	1,900 (21) [1,300–2,600]	63.8 [41.7–85.9]
	50–99	460 (7) [160–770]	14.6 [4.9–24.3]	630 (7) [330–940]	18.8 [9.7–27.8]
**Transmission category**	Male-to-male sexual contact	5,200 (75) [4,100–6,400]		6,800 (75) [5,400–8,200]*	
	Injection drug use	600 (9) [270–920]		940 (10) [500–1,400]	
	Male-to-male sexual contact and Injection drug use	280 (4) [80–480]		370 (4) [110–630]	
	Heterosexual contact^c^	830 (12) [370–1,300]		1,000 (11) [550–1,400]	
**Subtotal** c		7,000 [5,600–8,400]	41.2 [32.9–49.5]	9,200 [7,500–10,800]*	52.5 [42.8–62.2]
**Female**					
**Age at infection (yr)**	13–29	750 (37) [430–1,100]	12.5 [7.1–17.8]	780 (38) [390–1,200]	12.7 [6.3–19.2]
	30–39	580 (29) [280–890]	17.6 [8.5–26.7]	580 (28) [310–850]	17.0 [9.1–24.9]
	40–49	410 (20) [200–630]	15.4 [7.4–23.4]	440 (21) [170–710]	15.9 [6.2–25.5]
	50–99	270 (13) [20–520]	7.4 [0.4–14.3]	260 (12) [50–470]	6.7 [1.2–12.1]
**Transmission category**	Injection drug use	310 (15) [70–550]		330 (16) [80–570]	
	Heterosexual contact^c^	1,700 (85) [1,100–2,300]		1,700 (84) [1,100–2,300]	
**Subtotal** c		2,000 [1,400–2,600]	12.9 [9.0–16.7]	2,100 [1,400–2,700]	12.7 [8.7–16.8]
**Total** d		9,000 [7,400–10,600]	27.6 [22.6–32.5]	11,200 [9,300–13,100]*	33.4 [27.8–39.0]

		2008		2009	
		Incidence, No. (%) [95% CI]^b^	Rate [95% CI]	Incidence, No. (%) [95% CI]^b^	Rate [95% CI]
**Male**					
**Age at infection (yr)**	13–29	3,100 (43) [2,300–3,900]	43.0 [32.1–53.8]	3,000 (41) [2,300–3,800]	41.9 [31.5–52.3]
	30–39	2,500 (34) [1,800–3,200]	59.9 [42.5–77.3]	2,600 (35) [1,800–3,300]	60.6 [43.0–78.2]
	40–49	1,200 (16) [750–1,600]**	37.1 [24.0–50.3]	1,300 (18) [840–1,800]	40.8 [25.6–55.9]
	50–99	480 (7) [200–750]	13.3 [5.6–21.0]	460 (6) [200–730]	12.3 [5.2–19.2]
**Transmission category**	Male-to-male sexual contact	5,500 (76) [4,400–6,600]		6,000 (81) [4,800–7,100]	
	Injection drug use	680 (10) [300–1,100]		560 (8) [180–950]	
	Male-to-male sexual contact and Injection drug use	240 (3) [40–430]		230 (3) [40–410]	
	Heterosexual contact^c^	790 (11) [430–1,100]		640 (9) [350–930]	
**Subtotal** c		7,200 [5,900–8,500]**	40.0 [32.7–47.3]	7,400 [6,000–8,800]	39.9 [32.6–47.3]
**Female**					
**Age at infection (yr)**	13–29	730 (39) [380–1,100]	11.6 [6.0–17.1]	750 (37) [280–1,200]	11.6 [4.3–19.0]
	30–39	530 (29) [240–820]	15.3 [7.0–23.6]	560 (27) [240–870]	15.7 [6.9–24.6]
	40–49	440 (23) [200–670]	15.2 [7.0–23.5]	480 (24) [210–760]	16.4 [7.1–25.7]
	50–99	160 (9) [30–290]	4.0 [0.8–7.2]	240 (12) [20–450]	5.6 [0.6–10.7]
**Transmission category**	Injection drug use	280 (15) [80–480]		370 (18) [0–740]	
	Heterosexual contact^c^	1,600 (85) [1,000–2,100]		1,700 (82) [1,100–2,200]	
**Subtotal** d		1,900 [1,300–2,400]	11.1 [7.6–14.7]	2,000 [1,300–2,700]	11.8 [7.7–15.9]
**Total** d		9,000 [7,500–10,600]**	26.1 [21.6–30.6]	9,400 [7,800–11,000]	26.4 [22.0–30.9]

a Hispanics/Latinos can be of any race.

b CI, Confidence Interval. Confidence intervals reflect random variability affecting model uncertainty but may not reflect model-assumption uncertainty; thus, they should be interpreted with caution.

c Heterosexual contact with a person known to have, or to be at high risk for, HIV infection.

d Because column subtotals and totals for estimated numbers were calculated independently of the values for the subpopulations, the values in each column may not sum to the column subtotal or total.

*Indicates significantly different (p<0.05) from the 2006 estimate for the same group.

**Indicates significantly different (p<0.05) from the 2007 estimate for the same group.

**Table 4:** Estimated Number, Percentage, and Rate per 100,000 of New Infections with Associated 95% Confidence Intervals among Whites - United States, 2006–2009.

		2006		2007	
Characteristic		Incidence, No. (%) [95% CI]^a^	Rate [95% CI]	Incidence, No. (%) [95% CI]^a^	Rate [95% CI]
**Male**					
**Age at infection (yr)**	13–29	3,200 (23) [2,100–4,200]	14.4 [9.7–19.1]	3,900 (24) [2,600–5,200]	17.6 [11.8–23.3]
	30–39	4,700 (34) [3,400–6,000]	37.4 [26.7–48.0]	5,400 (34) [3,900–6,900]	43.5 [31.6–55.3]
	40–49	4,200 (31) [3,100–5,400]	27.0 [19.6–34.4]	4,600 (29) [3,400–5,900]	30.3 [22.2–38.3]
	50–99	1,700 (12) [1,100–2,400]	5.4 [3.4–7.3]	2,000 (13) [1,300–2,800]	6.2 [4.1–8.4]
**Transmission category**	Male-to-male sexual contact	11,700 (85) [9,500–13,900]		13,700 (86) [11,200–16,300]	
	Injection drug use	590 (4) [210–970]		650 (4) [210–1,100]	
	Male-to-male sexual contact and Injection drug use	880 (6) [380–1,400]		950 (6) [450–1,500]	
	Heterosexual contact^b^	600 (4) [180–1,000]		580 (4) [200–950]	
**Subtotal** c		13,800 [11,300–16,300]	16.8 [13.7–19.8]	16,000 [13,200–18,700]	19.3 [15.9–22.7]
**Female**					
**Age at infection (yr)**	13–29	970 (35) [380–1,600]	4.6 [1.8–7.4]	970 (32) [350–1,600]	4.6 [1.7–7.5]
	30–39	810 (29) [400–1,200]	6.6 [3.2–9.9]	880 (29) [450–1,300]	7.2 [3.7–10.7]
	40–49	660 (24) [320–1,000]	4.2 [2.1–6.3]	820 (27) [370–1,300)	5.3 [2.4–8.3]
	50–99	330 (12) [80–580]	0.9 [0.2–1.5]	320 (11) [100–530]	0.8 [0.3–1.4]
**Transmission category**	Injection drug use	860 (31) [310–1,400]		830 (28) [430–1,200]	
	Heterosexual contact^b^	1,900 (69) [1,200–2,600]		2,100 (72) [1,300–3,000]	
**Subtotal** c		2,800 [1,900–3,700]	3.2 [2.2–4.2]	3,000 [2,000–4,000]	3.4 [2.3–4.6]
**Total** c		16,600 [13,800–19,400]	9.8 [8.1–11.5]	18,900 [15,800–22,100]	11.2 [9.3–13.0]

		2008		2009	
		Incidence, No. (%) [95% CI]^a^	Rate [95% CI]	Incidence, No. (%) [95% CI]^a^	Rate [95% CI]
**Male**					
**Age at infection (yr)**	13–29	3,400 (27) [2,300–4,500]	15.2 [10.3–20.1]	3,700 (28) [2,500–4,800]	16.5 [11.3–21.6]
	30–39	3,900 (31) [2,800–4,900]	31.4 [22.6–40.3]	3,700 (28) [2,600–4,700]**	30.2 [21.4–39.0]
	40–49	3,600 (29) [2,600–4,600]	23.9 [17.4–30.5]	4,000 (30) [2,900–5,200]	27.5 [19.6–35.3]
	50–99	1,600 (13) [1,000–2,200]	4.8 [3.1–6.4]	1,900 (14) [1,200–2,600]	5.6 [3.6–7.6]
**Transmission category**	Male-to-male sexual contact	10,500 (85) [8,600–12,400]**		11,400 (86) [9,300–13,500]	
	Injection drug use	660 (5) [150–1,200]		640 (5) [110–1,200]	
	Male-to-male sexual contact and Injection drug use	550 (4) [210–880]		670 (5) [280–1,100]	
	Heterosexual contact^b^	670 (5) [220–1,100]		550 (4) [100–1,000]	
**Subtotal** c		12,400 [10,200–14,600]**	14.9 [12.3–17.6]	13,300 [11,000–15,600]	15.9 [13.2–18.7]
**Female**					
**Age at infection (yr)**	13–29	810 (34) [260–1,400]	3.8 [1.2–6.4]	740 (32) [280–1,200]	3.5 [1.3–5.7]
	30–39	740 (32) [350–1,100]	6.1 [2.9–9.3]	560 (24) [190–920]	4.6 [1.6–7.7]
	40–49	500 (21) [210–790]	3.3 [1.4–5.2]	710 (31) [220–1,200]	4.8 [1.5–8.2]
	50–99	300 (13) [100–510]	0.8 [0.2–1.3]	310 (13) [60–560]	0.8 [0.1–1.4]
**Transmission category**	Injection drug use	610 (26) [210–1,000]		650 (28) [180–1,100]	
	Heterosexual contact^b^	1,700 (74) [1,100–2,400]		1,700 (72) [920–2,400]	
**Subtotal** c		2,300 [1,600–3,100]	2.7 [1.8–3.6]	2,300 [1,500–3,200]	2.6 [1.7–3.6]
**Total** c		14,800 [12,300–17,200]**	8.7 [7.2–10.1]	15,600 [13,000–18,200]**	9.1 [7.6–10.6]

a CI, Confidence Interval. Confidence intervals reflect random variability affecting model uncertainty but may not reflect model-assumption uncertainty; thus, they should be interpreted with caution.

b Heterosexual contact with a person known to have, or to be at high risk for, HIV infection.

c Because column subtotals and totals for estimated numbers were calculated independently of the values for the subpopulations, the values in each column may not sum to the column subtotal or total.

** Indicates significantly different (p<0.05) from the 2007 estimate for the same group.

**Figure 2:**
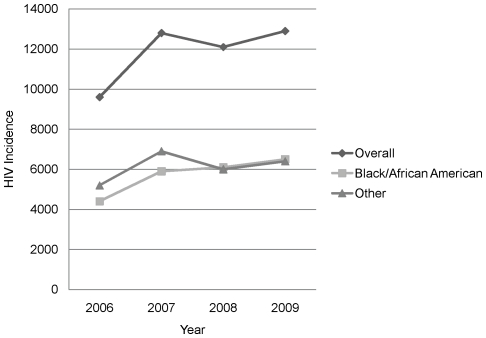
HIV incidence among 13–29 year old men who have sex with men (MSM) overall and by race/ethnicity – United States, 2006–2009. HIV incidence in all MSM 13–29 demonstrated a statistically significant estimated annual percentage change (EAPC) of 8.1% (95% CI: 1.9%–14.9%; p = 0.01). The EAPC for young black/African American MSM was 12.2% (95% CI: 4.2%–20.9%; p = 0.002) but was not significant for other young MSM.

Among the 13–29 year age group, by year, MSM made up 62%, 64%, 65%, and 69% of new infections, including 59%, 58%, 62%, and 66% of new infections among blacks/African Americans, 63%, 68% 65%, and 72% of new infections among Hispanics/Latinos, and 65%, 70%, 71%, and 72% of new infections among whites. Within MSM there were racial/ethnic differences in the age distribution of new infections. Among black/African American and Hispanic/Latino MSM, most new infections occurred in the youngest MSM, with MSM 13–29 accounting for 49%, 57%, 62%, and 60% of new infections by year among blacks/African Americans and 40%, 43%, 46%, and 45% of new infections by year among Hispanics/Latinos, compared with 23%, 25%, 28%, and 28% among whites (Table 5). Although there was a significant increase in new infections from 2006 to 2009 (34%, 95%CI: 8.4%–60.4%; p = 0.002) among young MSM overall (EAPC = 8.1%, 95% CI: 1.9%–14.9%; p = 0.01), the only significant increase in any MSM subgroup 2006–2009 was among young, black/African American MSM (48%, 95% CI: 12.3%–83.0%; p = 0.001), with EAPC 12.2% (95% CI: 4.2%–20.9%; p = 0.002). The EAPC among other young MSM was not significant (Figure 2). Among white MSM, the group most affected 2006–2008 was men 30–39 years of age, who accounted for 35%, 34%, and 31% of new infections by year and in 2009, men 40–49 years of age, who accounted 30% of new infections (Table 5).

**Table 5:** Estimated HIV Incidence and Associated 95% Confidence Intervals Among Black/African American, Hispanic/Latino^a^, and White Men Who Have Sex with Men, United States, 2006–2009.

		2006	2007	2008	2009
Characteristic		Incidence, No. (%) [95% CI]^b^	Incidence, No. (%) [95% CI]^b^	Incidence, No. (%) [95% CI]^b^	Incidence, No. (%) [95% CI]^b^
**Total (Black/African American)** c		9,000 [7,400–10,700]	10,400 [8,600–12,200]	9,800 [8,200–11,500]	10,800 [9,100–12,500]
**Age at infection (yr)**	13–29	4,400 (49) [3,500–5,400]	5,900 (57) [4,700–7,100]*	6,100 (62) [4,900–7,300]*	6,500 (60) [5,300–7,700]*
	30–39	2,300 (26) [1,600–3,000]	2,500 (24) [1,800–3,200]	2,000 (21) [1,400–2,600]	2,500 (23) [1,800–3,200]
	40–49	1,700 (19) [1,100–2,300]	1,400 (14) [960–1,900]	1,300 (13) [840–1,700]	1,400 (13) [870–1,800]
	50–99	560 (6) [230–880]	560 (5) [240–880]	430 (4) [190–670]	450 (4) [210–700]
**Total (Hispanic/Latino)** c		5,200 [4,100–6,400]	6,800 [5,400–8,200]*	5,500 [4,400–6,600]	6,000 [4,800–7,100]
**Age at infection (yr)**	13–29	2,100 (40) [1,400–2,800]	2,900 (43) [2,100–3,700]	2,500 (46) [1,800–3,200]	2,700 (45) [2,000–3,400]
	30–39	1,900 (36) [1,200–2,600]	2,300 (33) [1,600–3,000]	1,900 (35) [1,300–2,500]	2,000 (34) [1,300–2,700]
	40–49	1,000 (19) [530–1,500]	1,300 (19) [720–1,800]	790 (14) [450–1,100]	1,000 (17) [590–1,400]
	50–99	230 (4) [0–460]	360 (5) [140–590]	240 (4) [60–410]	300 (5) [90–500]
**Total (White)** c		11,700 [9,500–13,900]	13,700 [11,200–16,300]	10,500 [8,600–12,400]**	11,400 [9,300–13,500]
**Age at infection (yr)**	13–29	2,700 (23) [1,800–3,600]	3,400 (25) [2,300–4,600]	3,000 (28) [2,000–3,900]	3,200 (28) [2,200–4,200]
	30–39	4,100 (35) [2,900–5,300]	4,700 (34) [3,300–6,000]	3,300 (31) [2,300–4,200]	3,200 (28) [2,300–4,200]
	40–49	3,500 (30) [2,500–4,500]	3,900 (29) [2,800–5,000]	3,000 (28) [2,200–3,800]	3,400 (30) [2,500–4,400]
	50–99	1,400 (12) [850–1,900]	1,700 (13) [1,100–2,400]	1,300 (12) [810–1,700]	1,600 (14) [970–2,200]
**Total (All)** c		27,000 [23,000–31,000]	32,300 [27,800–36,800]*	26,900 [23,200–30,600]**	29,300 [25,400–33,200]
**Age at infection (yr)**	13–29	9,600 (36) [7,900–11,300]	12,800 (39) [10,600–14,900]*	12,100 (45) [10,100–14,100]*	12,900 (44) [10,800–14,900]*
	30–39	8,600 (32) [6,800–10,500]	9,900 (30) [7,900–11,800]	7,500 (28) [6,100–9,000]	8,000 (27) [6,400–9,500]
	40–49	6,500 (24) [5,100–7,900]	6,900 (21) [5,500–8,400]	5,300 (20) [4,100–6,500]	6,000 (21) [4,700–7,300]
	50–99	2,300 (8) [1,600–3,000]	2,800 (9) [1,900–3,600]	2,000 (8) [1,400–2,600]	2,400 (8) [1,700–3,100]

a Hispanics/Latinos can be of any race.

b CI, Confidence Interval. Confidence intervals reflect random variability affecting model uncertainty but may not reflect model-assumption uncertainty; thus, they should be interpreted with caution.

c Because column totals for estimated numbers were calculated independently of the values for the subpopulations, the values in each column may not sum to the column total.

*Indicates significantly different (p<0.05) from the 2006 estimate for the same group.

**Indicates significantly different (p<0.05) from the 2007 estimate for the same group.

Rather than expand the analysis to include in each year's analysis all areas that met the inclusion criteria for that year, we chose to limit the surveillance areas contributing data for analysis to those that met the inclusion criteria for *all* analysis years in order to ensure greater comparability across the analysis period. If we had expanded the analysis, the HIV incidence estimate for 2006 would have been higher by approximately 1.4% new infections and the estimates for 2007–2009 would have been lower than those presented by 2.3%%, 1.5%, and 3.1%respectively. Consistent with the analysis from 16 states and 2 cities, in the expanded analysis the only MSM subgroup to show a significant increase 2006–2009 was young, black/African American MSM (data not shown).

## Discussion

Based on the revised stratified extrapolation approach for estimating HIV incidence, the number of new infections in the United States remained relatively stable between 2006 and 2009. Our analysis examines HIV incidence over a four-year period to provide the clearest picture of the current status of trends in incidence. The only population with a change in HIV incidence over the entire four-year period was 13–29 year olds, and within that age group, the only risk group experiencing increases was MSM. Among young MSM the estimated number of new infections increased significantly from 2006–2009; the increase in incidence in this group was largely driven by a statistically significant increase in new HIV infections of 48% (12.2% annually) in young, black/African American MSM.

The point estimate of the number of new HIV infections in 2006 presented here is somewhat lower than the previous estimate but within the confidence interval of that estimate [4]. The difference is attributed to additional years of data including removal of duplicate case reports between states, and several improvements in the method, including calculating the probability of testing within the STARHS recency period using the observed testing frequency rather than the mean recency period which allowed us to mitigate the impact of artificially limiting the probability for repeat testers who test frequently. Additionally, using newly available information on the length and distribution of the STARHS recency period would be expected to decrease the estimate by approximately 4% even if no other revisions to the model had been made because the updated mean recency period is 4% higher than the mean recency period used for the previous estimate. Finally, uncertainty related to reporting delay adjustment may impact estimates. As additional years' data become available we expect that the HIV incidence estimates presented here will be revised further, consistent with estimates based on surveillance data which are subject to reporting delay. Because of anticipated adjustments to these estimates over time, it is important to focus primarily on the overall population impact and trends, rather than the precise point estimates, when drawing conclusions from these analyses.

Consistent with the higher rates of HIV diagnoses among MSM in general [13], and of both HIV diagnoses and HIV incidence in African American and Hispanic/Latino men and women [14]–[15], the 2006–2009 HIV incidence estimates continue to demonstrate the disproportionate impact of HIV disease in these groups. Although the results did not demonstrate a significant increase in HIV incidence among MSM overall, they indicate an increase among young people aged 13–29 that is largely driven by increases in incidence among young MSM, particularly young black/African American MSM. The increase in HIV incidence in young MSM is in line with the increase seen in new diagnoses in MSM in recent years in the United States [16] and internationally [17] as well as with increases in HIV incidence seen in the United States using an extended back-calculation model [4] and with international trends in incidence in MSM [18]–[19].

These estimates are subject to several limitations. First, in order to maintain consistency across the analysis years, we limited our analysis to only those states that consistently met the inclusion criteria for all analysis years. Therefore, the estimates are based on data from 16 states and 2 cities. The included areas represented 61% of reported cases of AIDS in the United States for the years 2006 and 2009, 62% for the years 2007 and 2008.

Because data were only available for a limited number of surveillance areas, we extrapolated our HIV incidence estimates from the included areas to the rest of the United States by applying the ratio of HIV incidence to AIDS incidence in the included areas to the AIDS incidence in the rest of the United States. To evaluate the validity of this extrapolation we compared the ratio of HIV diagnoses (as a proxy for incidence) to AIDS diagnoses in the included areas to that ratio in the areas to which we extrapolated. By extrapolating from the same surveillance areas each year, we may have underestimated HIV incidence overall by approximately 4%; using different areas each year, we may have underestimated HIV incidence by about 3%. Additionally, while the represented areas included several jurisdictions with very high morbidity, others—including the District of Columbia and the state of California—were not included in the estimate. Because HIV incidence in an area is driven by both risk behavior and HIV prevalence, the HIV incidence estimate may have been higher if these areas had been included. However, our method of extrapolating to the United States as a whole using the same 68 strata used for estimation likely compensates for the lack of inclusion of some cities with high HIV morbidity. It also limits overestimation of HIV incidence due to the differing ratio of HIV diagnoses to AIDS diagnoses in the incidence versus non-incidence areas.

Next, an assumption of the model is that HIV testing behavior has not changed over several years [4]. Comparing by year, the percentage of persons diagnosed with HIV and AIDS concurrently (within the same month), the percentage of persons classified as repeat testers, and among the repeat testers, the average time since last negative HIV test, the data indicate that HIV testing behavior remained stable over the analysis period. As a result of the apparent stability in these indicators of HIV testing behavior we did not adjust the model to account for a change in HIV testing behavior. CDC has recently funded HIV testing initiatives to reach individuals whose behavior puts them at high risk for HIV transmission and, in 2006, published recommendations for routine, opt-out testing for all individuals 13–64 seeking medical care [5]. While there are no data to indicate the level of uptake of the recommendations, evidence suggests that implementation of opt-out testing does increase HIV testing [20]. The data on history of HIV testing and antiretroviral use collected through HIV incidence surveillance may not have been collected over a long enough period of time to reflect recent changes in HIV testing behavior. If HIV testing has increased recently we may have overestimated HIV incidence in recent years. CDC will continue to track these indicators of testing behavior and adjust the estimation model as needed to reflect any changes detected in future years.

Additionally, concerns about using the BED assay within STARHS for cross-sectional estimation of HIV incidence have been raised for some HIV subtypes due to the misclassification of long-standing infections as recent [21], however, these issues are less relevant in the United States because of the predominance of HIV subtype B and as a result of the integration of STARHS results and supplemental data on history of HIV testing and antiretroviral use into national HIV surveillance [22]. This integration ensures that cases diagnosed with AIDS, at or within 6 months of HIV diagnosis, can be correctly classified as long-standing irrespective of STARHS result and allows for the removal and subsequent imputation of STARHS classification for cases in which antiretroviral use occurred prior to diagnosis. However, some HIV infections that were diagnosed late in the course of HIV disease, but not close to the time of AIDS diagnosis could have been misclassified as recent infections, adding to the uncertainty in calculation of HIV incidence and in comparison of HIV incidence across years.

In our previous work we noted that the HIV incidence estimate for 2006 could have been an overestimate if we had underestimated the likelihood of testing for HIV within one year of infection. The revised model for incidence estimation presented here addresses this concern by using the entire distribution of the STARHS recency period which limits the impact on the weights assigned to repeat testers who test more frequently than once per year, thus limiting the amount of overestimation bias inherent in the previous model. It is still possible, however that we could have overestimated incidence if a significant number of individuals were motivated to be tested for HIV by a real or perceived recent exposure, as this motivation was not addressed in the estimation model. We previously estimated that we may have overestimated HIV incidence by as much as 7% as a result of excluding motivation from the model to calculate incidence [4].

Finally, a number of additional assumptions of the model have been previously described [3]. These assumptions include that HIV incidence has been stable in recent years, that the likelihood of HIV testing prior to a diagnosis of AIDS is constant both with respect to time and to duration of HIV infection, that all HIV infections will eventually be diagnosed (either through testing or through death), and that individuals accurately report the HIV testing and treatment information, especially the date of their last negative HIV test. In addition, an assumption of using multiple imputation for missing data on BED result and HIV testing group (repeat or new testers) is that these data are missing at random, though not necessarily missing completely at random. These assumptions were addressed in the previous description of the model, and their effects were determined to be minimal, or to counterbalance one another.

The estimates for 2006–2009 continue to underscore the disproportionate toll that the HIV epidemic has taken on several populations in the United States including racial/ethnic and sexual minorities and injection drug users with 95% of new infections 2006–2009 estimated to have occurred in individuals in one or more of these groups. Though transmission rates have decreased substantially since the beginning of the epidemic [23], public health programs are presented with new challenges. There is a need to address the prevention needs both of people at risk for HIV infection as well as of those living with HIV disease—those who are aware of their HIV status and those who are not. With an estimated 21% of people living with HIV unaware of their HIV status [24], and the majority of new HIV infections transmitted by these individuals [25], it is important to expand testing to those people most at risk and provide them with care and prevention services. Adequate funding and services should be directed to individuals at greatest risk for acquiring and transmitting HIV infection, if we are to make a further impact on the HIV epidemic in the United States.

## Supporting Information

Table S1 **Probability of testing for HIV within the BED recency period (P) by number of months since most recent negative HIV test (T).**
(XLSX)

Table S2 **Group-level probability of testing for HIV within the BED recency period (P) based on the proportion of individuals diagnosed with AIDS at the time of HIV diagnosis (q).**
(XLSX)

Table S3 **Estimated diagnoses of HIV Infection, number BED tested, and estimated incidence of HIV infection in 16 states and 2 cities contributing data for analysis, 2006–2009.**
(XLSX)

## References

[1] Centers for Disease Control and Prevention (2010). HIV surveillance report, 2008. Vol. 20.

[2] Lee LM, McKenna MT (2007). Monitoring the incidence of HIV infection in the United States.. Public Health Rep.

[3] Karon JM, Song R, Brookmeyer R, Kaplan EH, Hall HI (2008). Estimating HIV incidence in the United States from HIV/AIDS surveillance data and biomarker HIV test results.. Stat Med.

[4] Hall HI, Song R, Rhodes P, Prejean J, An Q (2008). Estimation of HIV incidence in the United States.. JAMA.

[5] Centers for Disease Control and Prevention (2006). Revised recommendations for HIV testing of adults, adolescents, and pregnant women in health-care settings.. MMWR.

[6] Parekh BS, Kennedy MS, Dobbs T, Pau C, Byers R (2002). Quantitative detection of increasing HIV type 1 antibodies after seroconversion: A simple assay for detecting recent HIV infection and estimating incidence.. AIDS Res Hum Retrov.

[7] Parekh BS, Hanson DL, Hargrove J, Branson B, Green T (2010). Determination of mean recency period for estimation of incidence with the BED-capture EIA in persons infected with diverse HIV-1 subtypes.. AIDS Res Hum Retrov.

[8] Janssen RS, Satten GA, Stramer SL, Rawal BD, O'Brien TR (1998). New testing strategy to detect early HIV-1 infection for use in incidence estimates and for clinical and prevention purposes.. JAMA.

[9] Song R, Hall HI, Frey R (2005). Uncertainties associated with incidence estimates of HIV/AIDS diagnoses adjusted for reporting delay and risk redistribution.. Stat Med.

[10] McDavid Harrison K, Kajese T, Hall HI, Song R (2008). Risk factor redistribution of the national HIV/AIDS surveillance data: An alternative approach.. Public Health Rep.

[11] U.S. Census Bureau (2009). Population estimates: entire data set.. http://www.census.gov/popest/estimates.php.

[12] Kleinbaum DG, Kupper LL, Miller KE (1988). Applied regression analysis and other multivariable methods, 2^nd^ ed.

[13] Purcell DW, Johnson C, Lansky A, Prejean J, Stein R (2010). Calculating HIV and syphilis rates for risk groups: Estimating the national population size of men who have sex with men..

[14] Centers for Disease Control and Prevention (2007). Racial/ethnic disparities in diagnoses of HIV/AIDS—33 States, 2001–2005.. MMWR.

[15] Centers for Disease Control and Prevention (2008). Subpopulation estimates from the HIV incidence surveillance system—United States, 2006.. MMWR.

[16] Centers for Disease Control and Prevention (2008). Trends in HIV/AIDS diagnoses among men who have sex with men—33 states, 2001–2006.. MMWR.

[17] Sullivan PS, Hamouda O, Delpech V, Geduld JE, Prejean J (2009). Reemergence of the HIV epidemic among men who have sex with men in North America, Western Europe, and Australia, 1996–2005.. Ann Epidemiol.

[18] Le Vu S, Le Strat Y, Cazein F, Pillonel J, Brunet S (2010). Population-based HIV incidence in France, 2003–2008..

[19] Jansen I, Geskus R, Davidovich U, Coutinho R, Prins M (2010). Increasing trend in HIV-1 incidence among young men who have sex with men in Amsterdam: A 25-year prospective cohort study..

[20] Zetola NM, Grijalva CG, Gertler S, Hare CB, Kaplan B (2008). Simplifying consent for HIV testing is associated with an increase in HIV testing and case detection in highest risk groups, San Francisco January 2003–June 2007.. PLoS ONE.

[21] UNAIDS (2005).

[22] Centers for Disease Control and Prevention (2007). Using the BED HIV-1 Capture EIA Assay to estimate incidence using STARHS in the context of surveillance in the United States.. http://www.cdc.gov/hiv/topics/surveillance/resources/factsheets/BED.htm.

[23] Holtgrave DR, Hall HI, Rhodes PH, Wolitski RJ (2009). Updated annual HIV transmission rates in the United States, 1977–2006.. J Acq Immun Def Synd.

[24] Campsmith ML, Rhodes PH, Hall HI, Green TA (2010). Undiagnosed HIV prevalence among adults and adolescents in the United States at the end of 2006.. J Acq Immun Def Synd.

[25] Marks G, Crepaz N, Janssen R (2006). Estimating sexual transmission of HIV from persons aware and unaware that they are infected with the virus in the USA.. AIDS.

